# The use of photodynamic therapy in medical practice

**DOI:** 10.3389/fonc.2024.1373263

**Published:** 2024-05-08

**Authors:** David Aebisher, Kacper Rogóż, Angelika Myśliwiec, Klaudia Dynarowicz, Rafał Wiench, Grzegorz Cieślar, Aleksandra Kawczyk-Krupka, Dorota Bartusik-Aebisher

**Affiliations:** ^1^ Department of Photomedicine and Physical Chemistry, Medical College of The Rzeszów University, Rzeszów, Poland; ^2^ English Division Science Club, Medical College of The Rzeszów University, Rzeszów, Poland; ^3^ Center for Innovative Research in Medical and Natural Sciences, Medical College of The University of Rzeszów, Rzeszów, Poland; ^4^ Department of Periodontal Diseases and Oral Mucosa Diseases, Faculty of Medical Sciences in Zabrze, Medical University of Silesia, Zabrze, Poland; ^5^ Department of Internal Medicine, Angiology and Physical Medicine, Center for Laser Diagnostics and Therapy, Medical University of Silesia, Bytom, Poland; ^6^ Department of Biochemistry and General Chemistry, Medical College of The Rzeszów University, Rzeszów, Poland

**Keywords:** photodynamic therapy (PDT), medical practice, photosensitizers, treatment cancer, oncology

## Abstract

Cancer therapy, especially for tumors near sensitive areas, demands precise treatment. This review explores photodynamic therapy (PDT), a method leveraging photosensitizers (PS), specific wavelength light, and oxygen to target cancer effectively. Recent advancements affirm PDT’s efficacy, utilizing ROS generation to induce cancer cell death. With a history spanning over decades, PDT’s dynamic evolution has expanded its application across dermatology, oncology, and dentistry. This review aims to dissect PDT’s principles, from its inception to contemporary medical applications, highlighting its role in modern cancer treatment strategies.

## 1 Introduction

Photodynamic Therapy (PDT) is a treatment that uses a combination of light-absorbing photosensitizers (PS) and dissolved oxygen to kill diseased cells. Briefly, a patient is injected with PS and, after a period of time known as the drug-light interval, the diseased region is illuminated with a specific wavelength of laser light. Superoxide (O_2_^•-^) and hydroxyl radical (OH^·^), are the active agents responsible for eliciting cancer cell death as opposed to agents which are toxic to the body as a whole such as chemotherapy drugs and radiation. Photosensitizers- A photosensitizer is a chemical or compound that increases the sensitivity of the body or tissues to light, especially ultraviolet (UV) radiation or visible light. The effects of a photosensitizer may include hypersensitivity to light, allergic reactions or damage to skin cells under the influence of light radiation. For this reason, photosensitizers are used in photodynamic therapy. Reactive oxygen species- Reactive oxygen species (ROS) are chemically reactive species that contain oxygen in their structure. These types of compounds include peroxides, superoxide, hydroxyl radical and singlet oxygen. Immunogenic cell death (ICD) is a certain, regulated form of cell death that initiates innate and adaptive immune responses through molecular mechanisms.

The history of PDT began in 1900, when Raab (1) observed that microorganisms immersed in an acridine orange solution died after exposure to sunlight, while those immersed in pure water and exposed to such light still lived (2).

Since the late 1970’s, PDT is an attractive technology in cancer treatment. PDT is approved for the treatment of cancer in countries such as the United States, United Kingdom, France, Germany and Japan (3). The modern era of PDT was opened by the work of Lipson, which was associated with the isolation of a mixture of porphyrin derivatives, known in the literature as HpD (hematoporphyrin derivatives), from expired erythrocytic mass, which selectively accumulated in the tumor tissue and deposited there for several dozen hours. **Table 1** shows milestones of PDT.

**Table 1:** Milestones of photodynamic therapy (PDT).

Year	Milestone	Reference
**1976**	PDT in bladder cancer. First trial using hematoporphyrin in patients. Bladder cancer was diagnosed in 5 people, and tumor growth was slowed down in 1 person.	(4)
**1978**	PDT in skin cancer – a large-scale study.	(5)
**1979**	PDT in breast cancer.	(6)
**1980**	Bronchofiberoscopic PDT in early stage lung squamous cell carcinoma.	(7)
**1981**	First application of PDT concepts in the UK to patients with skin and ENT cancers.	(8)
**1982**	PDT in patients with tumors causing respiratory obstruction.	(9)
**1983**	PDT in esophageal cancer.	(10)
**1984**	PDT for endobronchial tumors. PDT shrinks tumors in the esophagus – both adenocarcinoma, squamous cell carcinoma, and melanoma.	(11, 12)
**1985**	PDT in dermatological and plastic surgery. The influence of external factors (temperature) on PDT effectiveness.	(13, 14)
**1986**	PDT in pelvic cancer in women.	(15)
**1987**	Theory of PDT dosimetry.	(16)
**1988**	Protocol for the treatment of bladder cancer.	(17)
**1989**	PDT in the recurrence of superficial bladder tumors after ineffective conventional treatment.	(18)
**1990**	First use of 5-aminolevulinic acid (ALA-PDT) in the treatment of actinic keratosis (AK), basal cell carcinoma (BCC) and squamous cell carcinoma. PDT in both early superficial esophageal cancer (ES) and advanced invasive cancer (AI).	(19, 20)
**1991**	PDT in rectal cancer. A protocol for measuring the clinical and radiological response in the above-mentioned tumor was established and a new system for administering light to the intestine was introduced.	(21)
**1992**	Combination of PDT and chemotherapy.	(22)
**1993**	Intraoperative PDT in the treatment of disseminated intraperitoneal tumors.	(23)
**1994**	Resection and intrapleural PDT in isolated pleural cancer. PDT in Bowen’s disease and in the treatment of basal cell carcinoma (BCC). PDT in the treatment of intraocular tumors.	(24–26)
**1995**	PDT in carcinoma *in situ* (CIS) of the entire bladder wall. PDT in the treatment of laryngeal cancer. PDT in AIDS-related cutaneous Kaposi’s sarcoma.	(27–29)
**1996**	Excimer dye laser instead of argon dye laser. PDT more effective than cryotherapy in Bowen’s disease. PDT in patients with malignant supratentorial glioma may prolong survival.	(30–32)
**1997**	PDT in patients with high-grade pleural mesothelioma (MPM).	(33)
**1998**	5-ALA-PDT in the treatment of oral leukoplakia.	(34)
**1999**	FDA officially approves ALA-PDT for the treatment of actinic keratosis (AK). PDT in the treatment of resistant warts on the hands and feet, as well as plaque psoriasis.	(35–37)
**2000**	PDT in pituitary adenomas infiltrating the dura.	(38)
**2001**	The European Union, Australia and New Zealand have approved methyl aminolevulinate (MAL-PDT) for the treatment of actinic keratosis (AK) and basal cell carcinoma (BCC). Principles of PDT for choroidal neovascularization (CNV) in age-related macular degeneration (AMD).	(39, 40)
**2002**	PDT in cardiology and cardiac surgery. The possibility of destroying atherosclerotic plaque using photodynamic therapy was presented. Moreover, it was noted that PDT may inhibit intimal hyperplasia, which results in vascular restenosis.	(40, 41)
**2003**	Japan has approved PDT using talaporfin sodium combined with a semiconductor laser for the treatment of early-stage lung cancer.	(42)
**2004**	PDT in patients with moderate to severe inflammatory acne. A new photosensitizer for PDT - talaporfin sodium - has been developed in Japan. PDT for interdigital mycosis caused by dermatophytes and yeasts. PDT in patients with breast cancer infiltrating the chest wall.	(43–46)
**2005**	PDT in limb salvage in musculoskeletal sarcomas. Visual rejuvenation of the face, especially the eye area, using 5-ALA-PDT.	(47, 48)
**2006**	ALA-PDT and MAL-PDT in the treatment of acne vulgaris. PDT in the treatment of genital warts in selected cases that do not respond to other therapies.	(49, 50)
**2007**	ALA-PDT is more effective, safer and simpler than CO_2_ laser therapy for genital warts. Beneficial effect in the treatment of aggressive periodontitis using PDT and in the treatment of squamous cell carcinoma *in situ* (CIS) of the penis.	(51–53)
**2008**	The anti-apoptotic properties of Bcl-2 are an important factor in PDT. The synergistic effect of photodynamic therapy and Bcl-2 antagonist has been proven.	(54)
**2009**	PDT in esophageal varices. PDT in the treatment of vulvar lichen sclerosus (LS).	(55, 56)
**2010**	Guidelines for the practical use of MAL-PDT in the treatment of non-melanoma skin cancer.	(57, 58)
**2011**	A robotic system that supports doctors in the even distribution of laser radiation in PDT in port wine stains (PWS).	(59)
**2012**	Daylight PDT as an alternative in the treatment of benign actinic keratosis (AK). Efficacy of MAL-PDT in the treatment of malignant glioma. Antibacterial potential of PDT in patients with chronic wounds.	(60–62)
**2013**	PDT in the treatment of actinic keratosis and the dermatological quality of life index (DLQI).	(63)
**2014**	Molecular mechanism of PDT in periodontal treatment. PDT in diabetic patients prevents amputation of the diabetic foot.	(64, 65)
**2015**	The thickness of the MAL layer does not significantly affect the accumulation of this photosensitizer in the treatment of basal cell carcinoma (BCC) and actinic keratosis (AK). The best way to anesthetize patients during PDT is scalp nerve blocks. Temoporfin is a better photosensitizer in photodynamic therapy of unresectable hilar cholangiocarcinoma than porfimer. An innovative high-resolution magnifying video endoscope enabling photodynamic diagnosis and PDT of stomach cancer.	(66–69)
**2016**	PDT combined with low-power laser therapy (LPL) and miconazole gel in the treatment of denture stomatitis. Pulsed PDT in the treatment of actinic keratosis (AK). The concept of sonodynamic therapy (SDT) as a new approach in non-invasive removal of solid tumors.	(70–72)
**2017**	Pretreatment with 5-fluorouracil cream increases the effectiveness of subsequent daylight photodynamic therapy for the treatment of actinic keratosis (AK).	(73)
**2018**	The effectiveness of the combination of 5-FU and PDT therapy is due to increased photosensitizer accumulation and p53 induction. A combination of both therapies was recommended. Efficacy of ALA-PDT in the treatment of vitiligo. A complication of ALA-PDT in the treatment of genital warts is more frequent infections among patients. Topical application of fusidic acid or mupirocin reduced the incidence of infection.	(74–76)
**2019**	Potential of EUS-PDT in the treatment of locally advanced pancreatic cancer (LAPC).	(77)
**2020**	The use of mTOR inhibitors in combination with PDT increases the therapeutic effect. Photodynamic therapy using a new laser probe - composite optical fiber (COF), which allows precise laser illumination of the cancerous lesion and its simultaneous visualization. The possible use of PDT in dermatology in Europe has been updated. The recommendations include such diseases as: actinic keratosis, superficial and nodular basal cell carcinoma, and field cancerization.	(78–80)
**2021**	5-ALA-PDT may be a new approach in the adjuvant treatment of glioblastoma than existing treatment regimens. The effectiveness of photodynamic therapy in addition to antiviral therapy for herpes labialis has been proven.	(81, 82)
**2022**	PDT with hypericin is effective in early-stage cutaneous T-cell lymphoma and has a favorable safety profile.	(83)
**2023**	PDT using methylene blue (MB) supports image-guided percutaneous abscess drainage.	(84)
**2024**	Intensive PDT with pulsed light (IPL-PDT) significantly inhibits seborrhea and improves the functionality of the skin barrier.	(85)

One of the milestones in the history of the development of the PDT method was the idea of local introduction into the body of the precursor of the actual sensitizer protoporphyrin IX (PPIX) - 5-aminolevulinic acid (ALA, aminolevulinic acid).

In 1990, Kennedy and Pottier first used ALA-based PDT (ALA-PDT) in a clinical setting, and since then ALA-PDT has seen rapid development.

The rapid elimination of excess this acid, as well as synthesized PPIX, from the body reduces the risk of photodynamic reaction of healthy skin to a minimum. After ALA administration, protoporphyrin IX accumulates mainly in the mucous membranes, not in the submucosa or muscles, which provides a chance for selective destruction of epithelial cells without affecting the supporting structures of the tissues.

5-Aminolevulinic acid can be administered orally or topically, unlike other sensitizers that are administered intravenously or intratissuely. In the 1980s, a number of dyes with photosensitizing properties were developed, such as porphyrin derivatives, chlorite and bacteriochlorins. They are derivatives of PPIX, which is a ligand for the red blood pigment - heme - and are characterized by a flat ring and the presence of conjugated double bonds.

However, due to the costs and side effects of PDT, i.e. untargeted light distribution, long half-life of metabolic clearance (almost 4–6 weeks), which significantly complicates its clinical application. Has lost its valuable role in clinical practice around the world (86).

Currently, many researchers are working on increasing the efficiency of PS, they believe that improving target specificity and increasing effective light penetration are key factors for the development of PDT-based anticancer photodynamic therapy (87).

According to the latest reports from the medical research, PDT can serve not only as a tool in the fight against cancer, but also lead to the elimination of pathogenic microorganisms (1, 88–92). This new type of therapy is called photodynamic inactivation of organisms (PDI) (93). Many diseases, especially dermatological, already have specific treatment regimens using the photosensitizer-light-oxygen system, but others still require further research, especially for clinical use (94). Despite the increasing understanding of the complexity of PDT process, including the activation of the patient’s immune system cells, some limitations of the therapy remain difficult to overcome (95, 96). For this reason, it is important to further expand the area of research on combining PDT with other types of therapy and the use of nanomedicine in drug delivery to the tumor area, as well as overcoming other existing problems that limit the effectiveness of PDT (95, 97). PDT is gaining in popularity among physicians although few laboratory PDT systems have made the transition to clinical use due to limitations such as the tissue penetrating ability of visible light (98). The increased popularity of PDT in the clinic is a case to perform more research on cellular mechanisms in response to PDT, for example, mitochondrial PD-L1 expression in tumors which is called a new window for mitochondrial immunotherapy for cancer patients (98).

## 2 Materials and methods

This review is based on materials and research found in the PubMed, ScienceDirect Google Scholar and ResearchGate databases published in the time period between 1970 and 2023. Search terms such as “PDT in medical practice,” “the use of PDT,” “photosensitizers in photodynamic therapy,” “the current status of PDT,” “PDT in dermatology,” and other analogous terms relating to possible applications of photodynamic therapy in practice in the past and currently were used in the search. Article selection was based on title, abstract, language and availability. Duplicate records were removed. Review and research articles as well as case reports were included.

## 3 Review of the literature

### 3.1 Basics of photodynamic therapy

**Figure 1:**
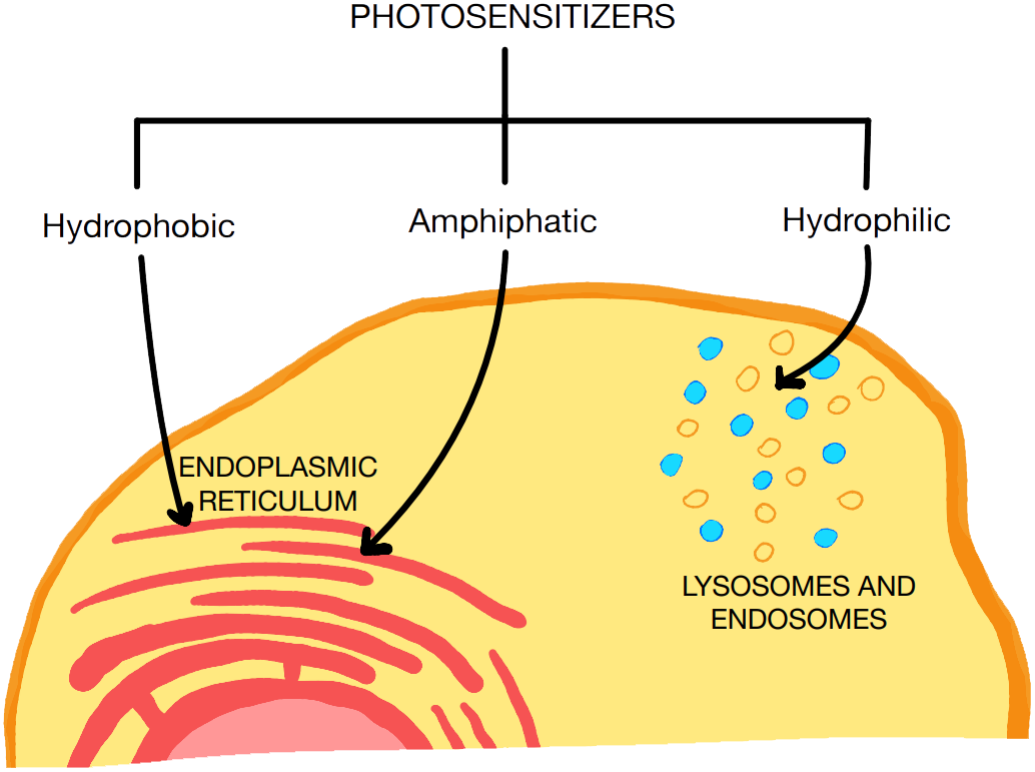
Types of PS used for PDT. PS with hydrophilic and amphipathic properties have a greater affinity for the endoplasmic reticulum than hydrophilic PS.

The photodynamic therapy (PDT) method, is a developing methodology of anticancer treatment. PDT uses photosensitizers (PS), which are injected intravenously or applied topically to achieve intracellular accumulation and then activated by light, which induces apoptosis, necrosis, immunogenic cell death (ICD), microcirculation damage and invokes a local immune response (90–98). The type of process resulting in cell death is primarily influenced by the location of accumulation of the PS. To activate the host’s immune system against cancer, it is most beneficial to initiate reactive oxygen species (ROS) formation in the endoplasmic reticulum (ER) to induce oxidative stress. For PS to accumulate in the ER, it should possess hydrophobic or amphipathic properties. Hydrophilic PS accumulate mainly in endosomes or lysosomes in the cytoplasm (99) (**Figure 1**). The formation of ROS in PDT can occur in two ways (Type I and II reactions). Type I reactions involve a direct interaction between the photoexcited dye and cellular oxygen resulting in the production of ROS. However, in Type II reactions, singlet oxygen (^1^O_2_) is formed as a result of energy transfer between the photoexcited PS and oxygen (100, 101). Both types of reactions can occur simultaneously, and which one prevails depends, among others, on the type, dose and physicochemical properties of the PS used (102). Photon absorption by PS can result in several deactivation processes. The first possibility is fluorescence emission from the excited singlet state. Secondly, after PS is excited to a singlet state, the PS can intersystem cross to the excited triplet state, which, when reacting with some endogenous substances, can create free radicals (O_2_^•-^). The third possibility is the transition of the excited singlet state to the triplet state and subsequent collision of PS with molecular oxygen and formation of ^1^O_2_; this pathway is frequently exploited in PDT (103, 104).

The effect of PDT is influenced by modifications of any of the PDT elements, such as the type of PS, light fluence, and dissolved oxygen concentration. Furthermore, the phenotypic variability of tumor cells and the environmental context of the tumor are determining factors (105).

**Figure 2:**
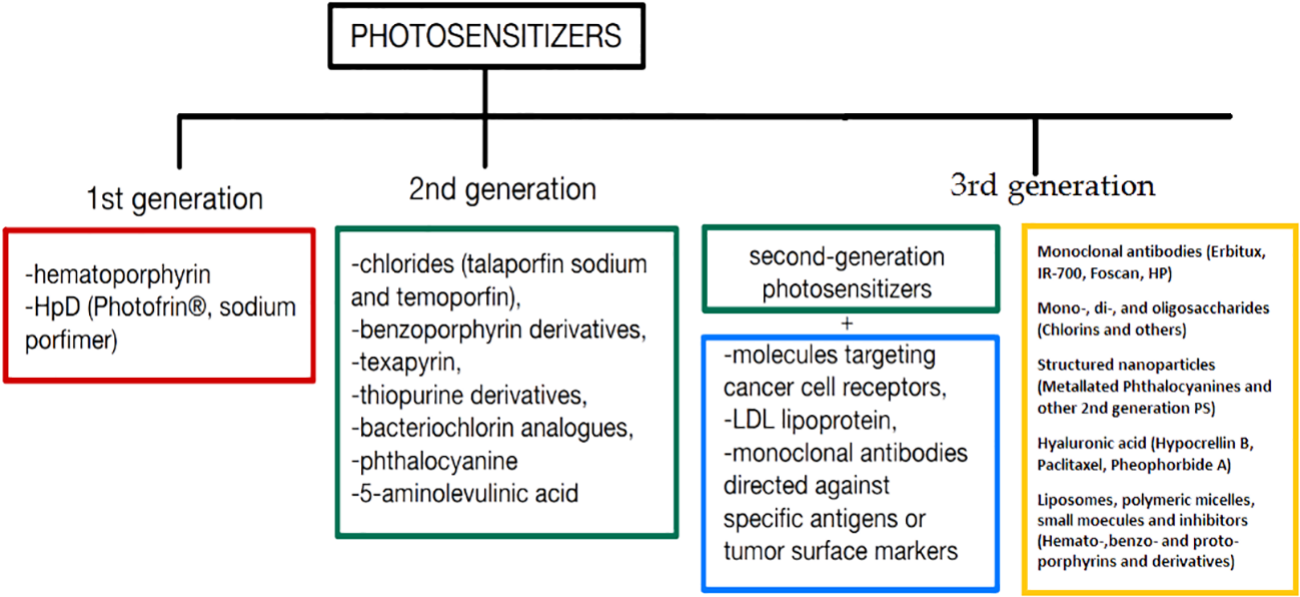
Classification of PS by generation, which differ in structure, selectivity of action, or accumulation in tissues.

The ideal PS should be characterized by the absorption of light at red or near infrared wavelengths and the ability to penetrate the blood-brain barrier (BBB). Moreover, it should have high selectivity towards cancer tissue, low dark toxicity and rapid clearance from the body (106). The BBB serves an important role by maintaining homeostasis and preventing macromolecules, infectious agents, and potential neurotoxins from entering the brain (107). However, the BBB also significantly limits the ability of therapeutic agents to reach their targets in the CNS. More than 90% of all small-molecule drugs and nearly 100% of all larger therapeutics are prevented from crossing the BBB. Photodynamic therapy (PDT) is a treatment that combines light irradiation and photosensitizing agents (porphyrins, chlorins and many other photodynamic dyes). The excited photosensitizer directly oxidizes biomolecules (Type I) and/or interacts with molecular triplet oxygen (^3^O_2_) producing singlet oxygen (^1^O_2_) (Type II) causing cancer cells apoptosis and/or necrosis through plasma and mitochondria membrane disrupture. It has recently been shown in several studies that PDT can temporally increase the BBB permeability. Hirschberg et al. using a 400 µm bare flat-end quartz fiber (635 nm) and stereotaxic procedure show targeted opening of the BBB for gadolinium (108). Madsen et al. using 670 nm laser application through the skull and aluminum phthalocyanine disulfonate (AlPcS_2a_) demonstrate the opening of the BBB for macrophages (109).The effectiveness of the therapy is directly proportional to the amount of singlet oxygen generated after using the optimal light wavelength (105). The first PS to be studied in detail was a derivative of hematoporphyrin (HpD; Photofrin^®^) (106). Currently, light-active molecules (PS) are divided into three generations of compounds. The first generation (**Figure 2**) includes naturally occurring porphyrins, such as hematoporphyrin and HpD (Photofrin^®^), a mixture of porphyrin dimers and oligomers, also known as sodium porfimer (110–112). The second generation (**Figure 2**) includes chlorins (talaporfin sodium and temoporfin), benzoporphyrin derivatives, texapyrin, thiopurine derivatives, bacteriochlorin analogues, phthalocyanine and the pro-drug 5-aminolevulinic acid (5-ALA) which [according to the latest data (110, 112)] are more effective in the production of singlet oxygen. Talaporfin sodium is one of the examples of photosensitizers belonging to the second generation group. The main advantages of these PS include: low toxicity and better selectivity during the process of destruction of cancer cells compared to first-generation photosensitizers (113). One example of the use of talaporfin sodium in the treatment of gastroenterological diseases is the use of PDT in the treatment of biliary tract cancer. Study results showed that PDT combined with the application of talaporfin sodium showed higher effectiveness in controlling tumor growth compared to PDT with the application of porfimer sodium (114). The third generation (**Figure 2**) of photosensitizing compounds are characterized by greater selectivity towards cancer cells and minimal accumulation in normal tissue. It includes combinations of second-generation photosensitizers with molecules targeting cancer cell receptors, combinations with LDL lipoprotein, monoclonal antibodies directed against specific antigens or tumor surface markers (such as growth factor receptors, transferrin or some hormones) (110, 112, 115).

Currently, the most commonly used PS’s are the prodrug 5-ALA which is enzymatically converted to protoporphyrin IV endogenously, and requires stimulation with a laser with a wavelength of 630-650 nm. Similarly methyl aminolevulinate (MAL), requires red light in the range of 570-670 nm (116). The light source may be suitably adapted continuous or pulsed lasers, optical fiber devices (made of quartz fibers with cylindrical tips or a lens), modern implantable devices, capsules and others (117, 118).

An interesting strategy for the near future is the development of multifunctional PS which activate after receiving a biological stimulus (e.g. temperature, pH, enzymatic activity) or physical stimulus (e.g. activation of an artificial factor, application of a magnetic or electric field, ultrasound, two-photon excitation), which could increase the selectivity of PDT. Examples include PS based on the peptide zipper principle, activated under acidic conditions, as well as the use of electroporation, which increases the transport of the photosensitizer to the pathological cell (93). Endothelial cells within the vascular system have the capability to concentrate PS, enabling them to generate free radicals upon activation by specific wavelengths of light. This phenomenon is crucial in PDT, where the vascular effect plays a significant role in the treatment’s overall efficacy. The effectiveness of PDT in targeting the vasculature can be notably enhanced by employing a short drug-light interval, which refers to the time between the systemic administration of the photosensitizer and the initiation of light exposure. This strategy ensures that the photosensitizer predominantly localizes within the vasculature, maximizing the vascular effects of PDT. Vascular PDT offers several advantages over PDT protocols that primarily rely on PS accumulation within tumor cells. Firstly, it involves the use of photosensitizers that are quickly cleared from the body, minimizing the risk of skin photosensitivity and other adverse effects associated with prolonged photosensitizer retention. Secondly, vascular PDT tends to exhibit higher long-term efficacy, potentially due to its ability to target the entire vasculature supplying the tumor, thereby impacting tumor growth and progression. Additionally, vascular PDT can often be completed in a single short session, offering convenience and efficiency in clinical practice. Overall, harnessing the vascular effect of PDT through optimized drug-light intervals and targeted endothelial cell activation holds great promise for enhancing the therapeutic outcomes of PDT, particularly in the context of treating vascular-related disorders and malignancies (119, 120).

PDT can induce immunosuppression, particularly in reactions to topical treatments with high fluence rates and large areas of irradiation. However, the oxidative damage inflicted by PDT on the tumor stroma leads to cell death, triggering an immune cascade. This cascade involves an acute inflammatory response initiated by the release of pro-inflammatory mediators, including cytokines, growth factors, and proteins. These mediators attract innate immune cells like neutrophils, mast cells, macrophages, and dendritic cells to the damaged tissue, aiming to restore homeostasis. Macrophages play a crucial role by phagocytizing PDT-damaged cancer cells and presenting tumor proteins to CD4 helper T lymphocytes, activating CD8 cytotoxic T lymphocytes. These cytotoxic T cells recognize and destroy tumor cells, promoting a systemic antitumor immune response. A promising strategy to enhance PDT selectivity involves developing activatable multifunctional photosensitizers that become active upon biological or physical stimuli. Biological stimuli include cancer-associated conditions like temperature, pH, and enzymatic activity. For instance, peptidic zipper-based photosensitizers are designed to react under acidic conditions. Electroporation is another effective method proposed to increase the transport of photosensitizers into pathological cells, potentially boosting cytotoxicity and PDT efficacy. Studies using different photosensitizers, including the clinically approved Photofrin, have shown promising results. Recent research suggests the use of transition metal coordination complexes or organic fluorophores as efficient photosensitizers for PDT. Transition metal complexes, such as ruthenium(II) and iridium(III) complexes, exhibit a heavy-atom effect facilitating interaction with molecular oxygen. They are easily synthesized and offer tunable properties. Organic fluorophores like naphthalimides, xanthenes, and boron dipyrromethene (BODIPY) can also serve as photosensitizers with high light absorption at long wavelengths, low toxicity, and good biocompatibility. Their fluorescence emission enables real-time monitoring during PDT treatment. In conclusion, PDT combined with electroporation shows promise in cancer treatment, but further studies are needed to elucidate its mechanisms fully. The development of novel photosensitizers with enhanced selectivity and efficacy holds great potential for improving PDT outcomes in cancer therapy (121–124).

### 3.2 PDT’s past

Already about 3,000 years ago, ancient inhabitants of Egypt, India and China used plant psoralen and sunlight to treat depigmentation lesions in vitiligo, as well as to treat psoriasis, rickets and skin cancer (89, 125). At the turn of the 19th and 20th centuries, Niels Finsen developed a field called “phototherapy.” He discovered that exposure to red light prevented the formation of smallpox pustules and was an effective therapy for treating the disease. He also used UV light to treat skin tuberculosis. In 1900, Oscar Raab, a medical student, noticed that certain wavelengths in the presence of acridine were lethal to a certain species of paramecium. In the same year, neurologist J. Prime stated that patients treated with oral eosin may develop local dermatitis located in the area exposed to sunlight. The breakthrough year was 1903, when Niels Finsen received the Nobel Prize, and independently, Herman Von Tappeiner and A. Jesionek used a combination of light and organic eosin dye in the treatment of skin cancer, which they called photodynamic action (89, 126). In 1913, Friedrich Meyer-Betz was the first to use hematoporphyrin on his own skin and observed swelling and pain, especially in places exposed to light (89, 127). In 1972, I. Diamond noticed that the phototoxicity of porphyrins could be used to kill cancer cells (89, 128). In 1975, Thomas Dougherty and colleagues reported that administration of hematoporphyrin in combination with red light completely inhibited mammary tumor growth in mice, and J. F. Kelly and colleagues reported that it also eliminated mouse urinary bladder cancer (89, 129, 130). In 1976, Kelly initiated the first trial using hematoporphyrin in patients, which successfully diagnosed bladder cancer in 5 people and slowed the growth of the tumor in 1. Moreover, it was observed that necrosis appeared in places exposed to irradiation (4, 89). In 1978, Dougherty conducted a larger study in which he used PDT on 25 patients with 113 skin tumors. Complete response to therapy was observed in 98 cases, partial response in 13, and 2 tumors were resistant (5, 89). Subsequent studies confirmed the effectiveness of PDT in esophageal, lung and stomach cancers (52, 53, 131).

### 3.3 Current applications of PDT

Over the years, many reports have been published about clinical trials involving the treatment of patients with oncological and non-oncological diseases using PDT. Clinical trials since the 1990s are briefly described below. Continuing research on the use of PDT in oncology is driven mostly by the search for an improved methods allowing for the efficient treatment of lesions at an early stage of cancer development. According to the Food and Drug Administration, PDT (132) is an approved treatment method for both dermatological diseases [advanced cutaneous T-cell lymphoma (133), basal cell carcinoma of the skin (134)], gastroenterological diseases (in diseases such as Barrett’s esophagus (135), esophageal cancer (136), and pulmonary diseases [cellular lung cancer (137, 138)]. Both experimental and clinical studies show that tissue-based PS accumulate in cancer cell organelles and vascular PS circulate in blood vessels. PDT can be performed on an outpatient basis. This reduces treatment costs and improves the patient’s mental comfort. The recent clinical progress of PDT in medical practice of cancer treatments showed that this method is limited by light penetration into tissue, oxygen dependency, photosensitivity followed by treatment, tissue oxygenation and lower effectiveness in metastatic cancers based on current technologies (139).

Healthcare providers utilize PDT to address a diverse array of medical conditions, encompassing skin ailments, various types of cancers, and certain noncancerous issues. PDT finds application in treating conditions such as skin cancer, lung cancer, esophageal cancer (including Barrett’s esophagus), bladder cancer, pancreatic cancer, bile duct cancer, head and neck cancer, as well as brain cancer. Following PDT, most individuals can promptly resume their daily activities, although some may require additional measures to safeguard their skin and facilitate healing in the treated area. To attain accreditation from the EURO-PDT Centre of Excellence, the unit underwent an audit by EURO-PDT, including a site inspection conducted by Professor Braathen. The inspection confirmed that the service met the highest standards. Established in 1986, the International Photodynamic Association (IPA) advocates for the scientific progression and clinical advancement of photomedicine, notably in photodynamic disinfection, photodynamic therapy (PDT), photoimmunotherapy (PIT), and photodiagnosis (PD). With members and affiliates spanning over 30 countries, the IPA represents a global community comprising distinguished international scientists, clinical and translational researchers, healthcare professionals, and students across various sectors. The IPA fosters the exploration of diagnosis and treatment through light-activated photosensitizers while disseminating scientific knowledge to its members, the research community, and the broader public. It organizes a Biennial World Congress, offering both members and non-members a unique platform to exchange insights and stay abreast of global developments and research in photodynamic therapy, photoimmunotherapy, and photodiagnosis.

**Figure 3:**
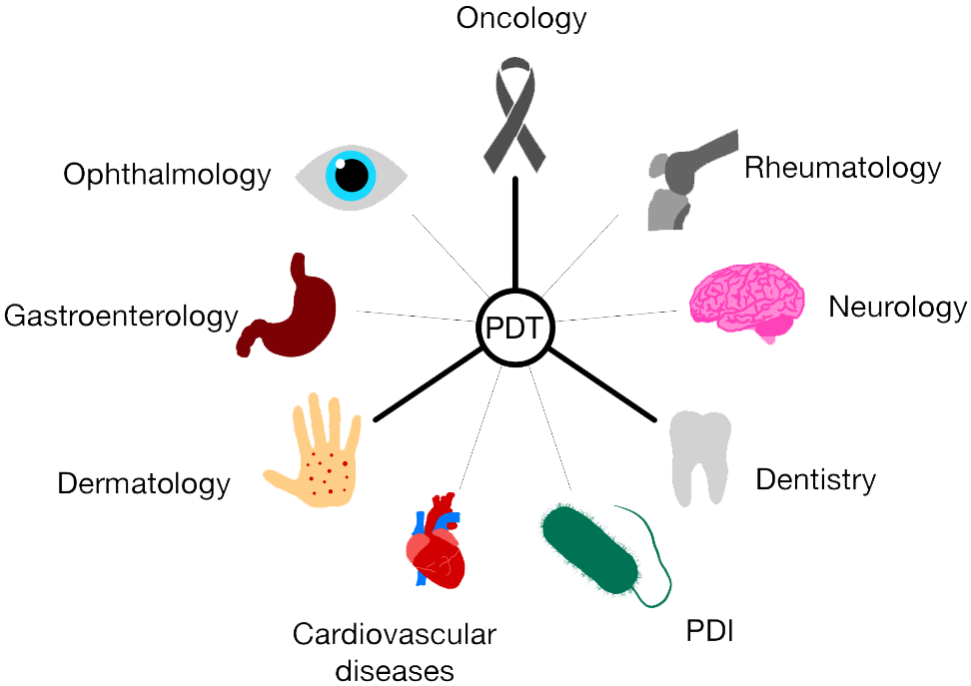
Application of PDT in various fields of medicine. The development of PDT is aimed at many different areas of medicine. Currently, the three most developed trends are those related to oncology, dermatology and dentistry. Photodynamic therapy is becoming increasingly important in ophthalmology, gastroenterology, cardiovascular diseases, neurology, rheumatology, and in combating bacteria, viruses and fungi thanks to photodynamic inactivation of microorganisms (PDI).

According to the latest reports, PDT is also being applied for the treatment of ophthalmological diseases (central serous chorioretinopathy, corneal neovascularization), cardiovascular diseases (atherosclerosis, esophageal varices), neurological diseases (Alzheimer’s disease), rheumatic diseases (rheumatoid arthritis), pulmonological diseases (cystic fibrosis) and gastrointestinal (Crohn’s disease) (93, 140). PDT is used primarily in dermatology, oncology and dental treatment (**Figure 3**) (90–92).

The invention of high-power light-emitting diodes, sLEDs, resulted in the selection of this type of sources for the PDT method as the most promising. sLED diodes combine the advantages of so-called LEDs and laser diodes, they are characterized by high light emission efficiency (1 W/A), high spatial coherence and low temporal coherence.

The device’s structure consists of three foldable, movable parts that enable the light-emitting surface to adapt to the shape of the body element. The illuminated surface depends on the device and its manufacturer, but is at least 20 cm^2^, and the head height is always adjustable, with the maximum adjustment being in the range of 102-182 cm. The device is equipped with 78 LEDs or more. The wavelength of the emitted radiation is approximately 630 nm (+/-5nm). The maximum radiation power density is 100–120 mW/cm². The device is operated via a touch screen. So far, it is the only device in the world that provides, among other things, a pulsed mode of operation and the so-called soft start, which means that during the first four minutes (1-4 min) the power increases during exposure from 40 to 100 of the maximum value. This is a very important advantage. This mode of operation protects against excessive decomposition of the photosensitizer in the initial period of exposure, and the pulsed mode ensures equalization of the level of oxyhemoglobin used during exposure to photooxidation of cellular structures. In clinical PDT, the radiation intensities acceptable to the patient are 50÷200 J/cm^2^, and the radiation density doses are 50-150 mW/cm^2^ for wavelengths of 630, 650 and 670 nm. In clinical conditions, the exposure time is 15–20 minutes and the light source should have a power of at least 1 W. For small surfaces, up to 2 cm^2^, sources with a power of 200-500 mW are sufficient, and a light power of 1.5-3 W is necessary to illuminate large surfaces. Generally, light sources used in the PDT method can be divided into laser and incoherent. Lasers are necessary to introduce light into the optical fiber and illuminate internal organs. For PDT purposes, radiation in the following wavelength range is practically used: — 390–410 nm — for photodiagnostics and therapy of super-surface lesions (e.g. acne); — 625–640 nm — for therapy (1st generation sensitizers: HpD, Fotofrin, PPIX); — 650–670 nm — for therapy (2nd generation sensitizers: chlorites, phthalocyanines. The output power of red light achieved through optical fiber reaches 2-3 W. Dye lasers pumped with the second harmonic of the Nd : YAG laser (Multilase Dye 600, Techno met Int.), copper and excimer lasers were also used. Currently, diode lasers have practically replaced other laser sources. They emit radiation with wavelengths from 375 nm to the near infrared (NIR) range. Halogen, xenon metal halide and mercury lamps were used as non-laser sources for the PDT method, mainly in laboratory conditions. The next step was to adapt them to clinical conditions and use high-brightness xenon lamps. Metal halide sources are very efficient and strong. Depending on the distance between the source and the illuminated surface, the irradiance varies from 30 to 240 mW/cm^2^, and the maximum illuminated surface is 300 cm^2^. A smaller, more labile metal halide source, with the possibility of changing optical filters and observing red fluorescence, was introduced into clinical practice (141). **Table 2** shows comparison of PDT with other commonly used therapeutic methods (142–149).

**Table 2:** Comparison of PDT with other commonly used therapeutic methods.

PDT	v.s. other techniques e.g. Surgery/Chemotherapy/Radiotherapy
**PDT efficacy**	PDT can only treat areas where light can reach. PDT can’t be used to treat cancers that have spread to many places.
**Side effect**	PDT has fewer side effects and toxicity than chemotherapy and/or radiotherapy
**PDT cost**	PDT is less than other cancer treatments. PDT can harm normal cells in the treatment area and cause side effects. The light used in photodynamic therapy can’t pass through more than about 1/3-inch of tissue, or 1 centimeter.
**patient quality of life**	PDT is non-invasive and non-surgical way of treatment with low toxicity Unlike radiation and chemotherapy, PDT can be repeated many times at the same site if needed.

Pain/burning is often felt during PDT, although its severity varies greatly. This symptom usually appears within minutes of the onset of light exposure and is thought to reflect nerve stimulation and/or tissue damage by reactive oxygen species, possibly exacerbated by overheating. Most patients tolerate PDT well without the need for anesthesia, but experiencing pain is more likely when larger areas of skin are treated, especially in well-nervated areas (face, scalp, hands, perineum), and more common during the removal of AK lesions than in Bowen’s disease. Patients with sensitive skin types seem to be more susceptible to pain. The second treatment in a two-treatment cycle may be more painful. It has not been possible to confirm that local anesthetics, tetracaine gel, a mixture of lidocaine 2.5% and prilocaine 2.5% or morphine gel significantly reduce pain during PDT. In a study on ALA-PDT for BD and BCC, anesthesia with a stream of cold air at -35° C shortened the duration of pain and the severity of pain, although cooling may slow down the photodynamic response. Transcutaneous nerve stimulation appears to have limited effect. Nerve blocks (e.g., forehead skin, entire scalp) are useful for treatments on larger surface areas and were superior to cold air anesthesia in a controlled half-face study of MAL-PDT in the treatment of multiple AKs in the frontal area. The PDT method using lower intensity light (daylight) is less painful, but the duration of therapy is longer. When using various pulsed light sources, PDT was associated with less pain compared to standard MAL-PDT. PDT often results in erythema and edema with erosions, crusting, and healing within 2–6 weeks, but ulceration is rare. After PDT, local photosensitivity may persist for 48 hours. Cases of post-inflammatory hypopigmentation and hyperpigmentation are rarely observed.

More commonly reported side effects include:

- Burning or stinging at the site of the photodynamic therapy treatment. This can be severe and may last 24-48 hours after your treatment.
- Redness and swelling of the treated area which may last up to 1 week
- Scaling or crusting of the treated area which may last up to 4 weeks

What limits side effects? To prevent a severe sunburn-like reaction you must strictly avoid direct sunlight, indirect sunlight through windows and skylights, and even bright indoor lighting for the next 48 hours. Sitting in a dimly lit room away from windows is best. Computer screens and televisions are okay. In addition, applying sunblock (containing either zinc oxide or titanium dioxide) to the treatment area every 2-3 hours during the day is strongly encouraged. Keep in mind however that sunblock will not fully protect the area from the sun if you were to go outside.

#### 3.3.1 PDT in dermatology

**Table 3** shows photosensitizers used in dermatology.

**Table 3:** PS in dermatology.

PS in dermatology	
**Photosensitizer**	**Current clinical state**
**5-ALA**	**clinical**
**ester methyl aminolevulinate (MAL)**	**clinical**

The skin is a place directly accessible and therefore extremely convenient for light irradiation (150, 151). Currently, PDT is approved in Europe for the treatment of actinic keratosis, squamous cell carcinoma *in situ*, superficial basal cell carcinoma, nodular basal cell carcinoma and the so-called field defect (field cancerization) (12). The local use of PDT in photorejuvenation and acne treatment seems promising, but optimization of treatment protocols is needed. Moreover, the use of PDT in the treatment of warts, vascular malformations, diabetic foot ulcers, hirsutism, keloid and alopecia areata seems to be promising (93, 140). Photodynamic therapy can also be considered in the treatment of Queqyrata erythroplasia, cutaneous T-cell lymphoma (CTCL), actinic cheilitis and some infections (cutaneous leishmaniasis, fungal infections, viral warts) (94, 150–152). PDT should not be proposed as a therapeutic option for psoriasis (94). The specific superficial location of skin cancer is a unique advantage and predisposition to the use of PDT (153). The PS’s used in dermatology are primarily 5-ALA or its ester methyl aminolevulinate (MAL). Three preparations are currently approved for use in Europe:

MAL Metvix ^®^ - used in combination with red light to treat non-hyperkeratotic actinic keratosis, Bowen’s disease and superficial basal cell carcinoma; 5-ALA Ameluz ^®^ - used in combination with red light in mild and moderate actinic keratosis (including those located on the face and scalp) and superficial basal cell carcinoma; 5-ALA AlaCare ^®^ - used in combination with red light in benign actinic keratosis. In North America, the 20% 5-ALA Levulan ^®^ preparation has been approved and is used in combination with blue light to treat actinic keratosis (93, 94). In clinical practice, a typical protocol for topical PDT application begins with lesion preparation by gently removing crusts and scales with a scalpel or curette, thoroughly washing the treated area with soap and water, and then removing any debris using a gauze pad soaked in acetone or 70% isopropylene alcohol. Then, apply two layers of photosensitizer evenly to the treated area. After applying the first layer, waiting until it dries, and after applying the second layer, the photosensitizer to left to incubate for 0.5-4 hours. Then, the PS is activated with an appropriate light source, and after the process, the treated area is washed with soap and water. The patient should be instructed to avoid sunlight for the next 2 days. The standard treatment regimen for AK includes one treatment, while in the case of BCC and Bowen’s disease - two treatments are applied spaced 7 days apart. If necessary, subsequent PDT sessions can be repeated after 2-3 weeks (93, 94). Below are examples of selected dermatological diseases in which PDT seems particularly interesting:

- Actinic keratosis (AK) is the most common precancerous lesion among Caucasians. Hyperkeratotic lesions appear in places exposed to the sun, such as the face, ears, forearms, and hands. Over time, they may transform into squamous cell carcinoma, especially when they are characterized by abundant infiltration, rapid enlargement, inflammation, diameter exceeding 1 cm, erythema, bleeding and ulceration (74–79, 117). PDT is currently approved for the treatment of AK in the USA, Canada and the European Union, but it has not yet been determined which photosensitizers have the best effect. Most sources offer classical 5-ALA-PDT or MAL-PDT as PDT therapy suggestions (74, 90, 154–164). Local PDT should be offered especially in the case of cosmetically sensitive areas, multiple and large lesions, and also in the case of patients with residual lesions in whom AK lesions showed a good response to previous treatment. The use of PDT should be considered in mild or moderate lesions when they affect an area where therapy may prove particularly painful, as in the case of confluent AK areas on the face or scalp (94). Photodynamic therapy also proven effective in preventing AK after solid organ transplantation (165). The improvement of the therapeutic effect of AK may be the result of combining PDT with the use of calcipotriol or 5-fluorouracil, which results from the increase in the level of PpIX (166, 167). Moreover, administration of high doses of vitamin D3 (10,000 IU daily for 5-14 days) significantly improves the response rate of AK treatment using PDT from 54.4% to 72.5% (168). Combining PDT with local treatments, microneedling, cryotherapy and laser therapy also improves the effect of therapy (169–172). In 1996, another paper was published presenting the results of a clinical trial by Szeimies et al. In this study, patients with a dermatological AK condition were treated. The aim of this study was to evaluate the effectiveness of PDT in combination with 5-ALA. The results were assessed by observing changes in the skin in relation to the grading scale. The clinical trial confirmed the effectiveness of PDT with 5-ALA in the local treatment of AK. Due to its relatively easy application, it is one of the key therapeutic methods in this type of dermatological disease (173). In 2007, Christiansen et al. conducted research on PDT with 5-ALA of the skin (174). In 2019 Osiecka et al. showed that PDT with green light for AK has similar efficiency as compared to red light with the use of 5-ALA (175). PDT is also one of the therapeutic methods for treating cheilitis. In a study conducted by Stender and Wulf, three patients diagnosed with cheilitis were included. The photosensitizer used was 5-ALA. The results clearly confirmed that the therapy was effective. Additionally, after several months of follow-up, no remissions were observed (176).
- Basal cell carcinoma (BCC) is the most common malignant skin tumor originating from immature, multidirectional differentiated cells, imitating early fetal forms of skin adnexa (177). In the case of the nodular type of this cancer, surgical treatment is recommended, especially in high-risk areas, while in other types of BCC, non-surgical treatment is often recommended, including PDT, the undoubted advantage of which is a shorter treatment time than other therapies and a visually better cosmetic effect (178–180). PDT is especially recommended as a treatment option for superficial, poorly healing BCC located in cosmetically sensitive places, in multiple and large lesions, as well as in patients with residual lesions that remain after previously effective treatment (94). Compared to treatment with imiquimod or 5-FU, PDT shows fewer side effects (104). ALA-PDT and MAL-PDT show similarly good effectiveness (181). It is recommended to use red light (90). The use of hexyl aminolevulinate (HAL) as a photosensitizer may also seem to be an interesting alternative (182). Pretreatment with CO_2_-assisted ablative fractional laser (AFL) before MAL-PDT is associated with less painful BCC therapy, with maintained efficacy and comparable side effects to traditional PDT (183). PDT has been described in the treatment of BBC with complete cure achieved using 20% 5-ALA (19). In 2007, Surrenti et al. performed a PDT study using methyl aminolevulinate (MAL-PDT) in the treatment of superficial and nodular BCC in sixty nine patients. The results were positive and BCC treatment with the MALPDT method showed high efficiency (184). In 2015, Haak et al. described studies of PDT involving ablative fractional lasers (AFXL) and methyl aminolevulinate (MAL) compared to conventional PDT for nodular basal cell carcinomas (nBCC) (185).
- Squamous cell carcinoma (SCC) *in situ*, also called Bowen’s disease, is a pre-invasive malignancy arising from epidermal keratinocytes in places exposed to UV radiation (especially in the head and neck). The complete response rate to PDT in the treatment of SCC is 82% (186–188). Due to poor tissue penetration by light and photosensitizer, PDT is more suitable for isolated, small, superficial SCCs (189). In addition, increased glycolysis and decreased oxidative phosphorylation, i.e. the so-called The Warburg effect plays a key role in SCC resistance to PDT. However, pretreatment with metformin may effectively increase the effectiveness of PDT (190). Similarly, the use of 5-FU may support ALA-PDT by increasing the amount of PpIX in SCC (191). Evidence for the prevention of SCC with PDT remains limited, however, the benefits of PDT clearly outweigh the losses, therefore, especially in the case of poorly healing, cosmetically sensitive skin areas, multiple and large lesions, the use of photodynamic therapy is recommended (94, 150). In microinvasive squamous cell carcinoma, photodynamic therapy should be considered as a treatment option when surgery is contraindicated. However, PDT should be avoided in the treatment of invasive SCC (94). In 2004, Rossi et al., conducted a study in which SCC carcinoma was treated using PDT with 5-ALA (192).
- Extramammary Paget’s disease (EMPD) is a rare intraepithelial tumor, accounting for approximately 1% of anogenital malignancies. The gold standard treatment for EMPD is surgery with a safety margin, but in the case of advanced tumors, other methods of therapy prove to be extremely useful (193). Compared to surgical methods, PDT in the treatment of EMPD shows a lower incidence of relapses and functional sequelae (194). In this case, ALA-PDT and MAL-PDT are also used (195). To improve the effectiveness of treatment, PDT therapy can be combined with, among others, a holmium laser or imiquimod (196–199). However, PDT is not a strongly recommended therapeutic option for EMPD (94).
- Seborrheic keratosis (SK) is a benign tumor with a small chance of developing into malignant squamous cell carcinoma. For this reason, it rarely requires any treatment. It often appears on the head and face, especially in middle-aged and older people (200–202). Two cases of the use of photodynamic therapy in SK have been described in the literature. The first one is a 61-year-old woman with a SK measuring 4.0 cm × 4.5 cm on the scalp. After ALA-PDT therapy, the lesion regressed significantly (202). The second case was an 81-year-old patient with SK and SCC on the scalp. In a man, a CO_2_ laser was successfully used three times, followed by ALA-PDT, and no recurrence was observed for 1 year (203).
- Acne vulgaris is a common dermatological disease associated with overproduction of sebum, disturbances in the process of keratinization of the sebaceous glands and colonization of hair follicles by Propionibacterium acnes bacteria. It mainly affects teenagers and young adults, but acne is more common in adults. Standard acne treatments include topical benzoyl peroxide, azelaic acid, retinoids and antibiotics (such as erythromycin, clindamycin), as well as antibiotics in general (especially tetracyclines), hormonal contraception and, finally, isotretinoin (204). However, it turns out that photodynamic therapy can be used as a treatment against this disease. Photodynamic therapy promotes antimicrobial and anti-inflammatory effects, increases epidermal exfoliation and inhibits the sebaceous glands, which may contribute to the inhibition of acne (205). After analyzing many studies, it was concluded that high doses of ALA-PDT and MAL-PDT produce similar effects with at least 3-hour incubation. Compared to blue and pulsed light, red light was found to be more conducive to the destruction of sebaceous glands (150). The combination of ALA-PDT with minocycline also seems interesting (206). In 2019, Serini et al. used 5% 5-ALA in the treatment of acne vulgaris in 35 patients. The published study showed the optimization of used concentration (207).
- Warts are a group of clinically diverse skin diseases caused by human papillomaviruses (HPV). Available therapeutic methods turn out to be ineffective and are associated with a tendency to relapse (36). Numerous randomized trials show the effectiveness of PDT in the treatment of refractory warts on the hands and feet, which is higher than placebo or cryotherapy (208–215). However, the lack of optimization of protocols and the pain occurring during treatment mean that doctors rarely prescribe PDT for the treatment of warts (216). Warts are another example of a non-malignant but aesthetically significant dermatological problem. One of the first studies on the treatment of this type of disease using PDT was developed by Stender et al. The topically applied PS was 5-ALA. A follow-up examination was performed 1 year after the therapy. According to the authors, the therapy was effective, and no recurrence of skin lesions was observed (208). In Poland, PDT research with azone (1-dodecyl-azepan-2-one) application prior to the application of 5-ALA in patients with mosaic and myrmecia plantar warts conducted. The results showed that pre-treatment with azone significantly increases the penetration of 5-ALA in tissues, and thus increases the effectiveness of PDT (36, 209, 217).
- Genital warts are one of the most common sexually transmitted diseases, and infection with the oncogenic type of HPV virus is the most important risk factor for the development of cervical cancer (218). There are many case reports and several larger studies that confirm the positive impact of PDT in the treatment of genital warts. In a study of 164 patients with urethral condylomas, as many as 95% were cured after one to four ALA-PDT treatments (51). It turns out that ALA-PDT is almost as effective as CO_2_ laser (95% to 100%), with a lower recurrence rate with photodynamic therapy (6.3% to 19.1% or 9% to 17%) (219, 220).

Using 5-ALA in the treatment of Bowen’s disease showed an 88% rate of complete cure (221). In 2005, Nybaek and Jemec Denmark conducted a study in which they used PDT to treat rosacea in 4 patients. Their results have shown that PDT may play a role in the treatment of selected cases of rosacea (217). In 2007, Berking et al. evaluated the efficacy of PDT in actinic cheilitis of the lower lip in 13 patients (222). In 2008, PDT treatment with HAL in cervical intraepithelial neoplasia (CIN) and human papillomavirus (HPV) infection have shown that HAL-PDT was performed (223). PDT treatment with the use of 16% and 8% methyl aminolevulinate (MAL) with exposure to daylight was found to be an effective and well-tolerated AK treatment (216). In 2012, research in Poland was carried out to assess the effectiveness of local PDT in basal cell carcinoma in 34 patients. PDT with Levulan resulted in a complete cure of 75% of patients (224). In 2014, a Polish research team from Białystok evaluate the clinical effectiveness of PDT using the Photolon photosensitizer (225). In 2017, researchers from Wrocław Poland successfully used PDT to a group of 11 patients with lichen sclerosus (LS) (226).

#### 3.3.2 PDT in oncology of internal organs

**Table 4** shows photosensitizers used in oncology of internal organs.

**Table 4:** PS application in oncology of internal organs.

PS application in oncology of internal organs	
**Photosensitizer**	**Current clinical state**
**Foscan**	**clinical**
**Photofrin**	**clinical**
**5-ALA**	**clinical**
**ester methyl aminolevulinate (MAL)**	**clinical**

PDT is increasingly used in the treatment of various solid tumors, including the brain, head and neck, lungs, gastrointestinal tract, bones, urinary bladder, prostate, breast, cervix and ovary (93, 227, 228).

##### 3.3.2.1 PDT in head and neck cancers

In 1996, a paper was published describing several years of randomized clinical trials using PDT in patients with head and neck cancer. The effectiveness of therapy and treatment results were assessed based on histopathological analysis of fragments taken during a follow-up biopsy. After analysis, it was found that 89% of patients had a complete and fully confirmed positive response to treatment (229). PDT in the treatment of head and neck cancer concerns treatment of recurrent leukoplakia and papilloma with the use of the photosensitizers Foscan and Photofrin^®^ (230). Experimental PDT has explored boronated porphyrins, which have the ability to cross the blood-brain barrier (231).

##### 3.3.2.2 PDT in cerebral glioma

Cerebral gliomas account for approximately 77% of all brain tumors and are the most common primary malignancies in the central nervous system (232). The World Health Organization (WHO) classification allows for dividing gliomas into four stages: I, II, III and IV. The first two grades, grade I and II gliomas, are considered low-grade gliomas (LGG) and include various subtypes of astrocytomas (including pilocytic and diffuse) and oligodendrogliomas. The group of grade III and IV gliomas, which are high-grade tumors (HGG), includes, among others: anaplastic astrocytoma and GBM (233). Although they do not occur frequently, they are characterized by a rapid growth rate, the ability to undergo angiogenesis and the ability to infiltrate adjacent tissues, which results in significant difficulties in achieving complete tumor resection (232, 234). Moreover, the presence of blood-brain and blood-tumor barriers limits the possibility of metastasis, while significantly complicating the therapy of these tumors. The average survival period is between 14.6 and 16.7 months. 90% of patients experience a relapse of the disease, and after a relapse the average life expectancy is 6.2 months (232, 235). In 1980, a pioneering photodynamic therapy for brain gliomas was introduced by Perria et al. As a complement to the surgical procedure, they used PDT, using hematoporphyrin as a photosensitizing substance and subjecting the remaining tumor bed to a helium-neon laser (632.8 nm) (235). Subsequent studies confirmed the beneficial effect of PDT on brain glioma. The studies used various combinations of light wavelengths and photosensitizers, including: the previously mentioned 5-ALA, as well as temoporfin (5,10,15,20-tetra(m -hydroxyphenyl)chlorin, which is the active ingredient of the EU-approved Foscan^®^ preparation), Photofrin ^®^, hypericin (HY), talaporfin and others. Despite the advancement in therapeutic approaches, currently no standard treatment options have been established in the event of glioma recurrence. There have been numerous preclinical and clinical studies examining the use of PDT in other common CNS malignancies, including meningiomas, pituitary adenomas, pediatric brain tumors, chordomas, chondromas, and others. The influence of both first and second generation photosensitizers was tested. Most studies have confirmed the significant effectiveness of the therapy, but further studies are necessary to better define the effectiveness of PDT (116, 236, 237). In Germany, in 2007, Stepp et al. used PDT for malignant glioma under fluorescence control (238). In 1996, Muller and Wilson used PDT to treat patients with newly diagnosed malignant supratentorial gliomas. Intravenous porphyrin PS was administered prior to surgery and photoillumination (32). In 2000, Krishnamurthy et al. showed that PDT in the treatment of brain malignancies in 18 patients (12 patients with glioblastomas, 5 patients with anaplastic astrocytoma, and 1 patient with malignant ependymoma) showed that increasing the light dose did not significantly improve the efficacy of PDT. Four patients had longer survival after using PDT (239). The Perrira Group described research using a device that allows percutaneous treatment of robotic malignant brain tumors by using optical fibers to induce PDT process (240). In 2013, Johansson et al. conducted a fluorescence and photobleaching study during interstitial PDT with protoporphyrin IX in gliomas (241).

##### 3.3.2.3 PDT in lung cancer

In turn, Hugh-Jones and Gardner were among the first to apply PDT to patients diagnosed with bronchial cancer. The photosensitizer used was hematoporphyrin or its derivatives. In 80% of patients treated, the tumor responded positively (242).

Another photosensitizer used in the treatment of gastroenterological and pulmonary diseases is sodium porfimer. An example of its use is lung cancer (243). In turn, Patelli et al. proposed a model for treating bronchial cancer using PDT. The research group included 23 patients. The authors clearly stated that PDT is effective in superficial bronchial lesions (244). In turn, Messmann et al., conducted a pilot study of the treatment of esophageal cancer in patients using PDT supported by hematoporphyrins. The study experimentally used laser light fractionation, which involves the application of a laser and delivery of light to the deep layers of the skin where many points of micro-damages are created, stimulating strong regenerative processes. A study by Messmann et al. showed that fractionation treatment improved the effectiveness of the therapy. According to the authors, this type of treatment may be an alternative technique to prevent the application of high doses of photosensitizers (245). Lung cancer is a high-grade cancer. The mortality rate from lung cancer reached 22.24% in Poland in 2020, which ranks it in the inglorious first place among all types of cancer (246). According to research, both PDT alone and its combination with chemotherapy have a beneficial therapeutic effect (247). In the case of early-stage non-small cell lung cancer (NSCLC), PDT is used as an endobronchial therapy to cure tumors growing in the bronchi. The situation is similar with multiple primary lung cancers. In advanced or metastatic NSCLC and small cell lung cancer (SCLC), PDT is used to relieve symptoms resulting from bronchial obstruction, such as pain, shortness of breath or hemoptysis. In advanced NSCLC, PDT is also used as a component of combined therapy with surgical resection. The light required to activate photosensitizers usually penetrates 5-10 mm into the tissue. For this reason, PDT is most often used to treat superficial tumors that affect the lining of organs or cavities. In tumors growing inside the bronchi, the light source is a laser directed endoscopically through fiber-optic cables to the lungs (248). Takita and Dougherty used PDT to treat malignant pleural mesothelioma. In this study, the therapeutic modality was endovascular PDT. As before, the PS used was Photofrin. After the treatment, the average survival time was 1 year (249). Taber et al. conducted a study in 1998 in which PDT was used to palliation of chest wall recurrence in patients with breast cancer. Seven patients with breast cancer were treated using PDT. Research results have shown that PDT is an effective treatment for chest wall recurrence in patients with breast cancer in whom other treatments have failed (250). In 2013, Bombeccari et al. conducted a study in which they used PDT to treat per-implant inflammation. In addition, patients treated with the PDT method had a lower pro-inflammatory index after 24 weeks of follow-up (251).

##### 3.3.2.4 PDT in gastrointestinal cancer

Gastrointestinal cancer is one of the main causes of death in developed countries, and in many cases the development of the disease is caused by an incorrect diet (252). PDT has been approved as a treatment for esophageal cancer. The use of PDT seems to be particularly important in superficial cancer lesions of the esophagus, which are difficult to treat using endoscopic resection, and also as a method used after local failure after radiotherapy, if the use of other therapeutic methods is difficult or impossible (253). Another indication for the use of PDT is Barrett’s esophagus, which is classified as a precancerous lesion. In the case of advanced cancer, PDT works as a palliative treatment method (86). Thanks to recent discoveries, using talaporfin sodium and diode laser, photodynamic therapy can be safely and successfully used in the treatment of esophageal squamous cell carcinoma (ESCC) (91). PDT is also used in other gastrointestinal cancers - including stomach, bile duct, rectal and pancreatic cancer (254). In turn, Harlow et al., conducted research on the treatment of rectal cancer using PDT. The research group included 22 patients. The photosensitizer used was Photofrin. The therapeutic method was intraoperative PDT. Due to the time of the research and its initial stages, the authors concluded that this type of therapy is not effective enough (254). Nseyo conducted a clinical trial on patients diagnosed with bladder cancer. Similarly to the previous studies, Photofrin was used as a photosensitizer. The study confirmed the effectiveness of PDT (255). Weber et al. used PDT in the treatment of gastrointestinal cancer. The photosensitizer used was 5-ALA. The effectiveness of the therapy was measured using fluorescence. The experimental results confirmed the effectiveness of PDT (256). In 1991 Muller and Wilson conducted a pilot study to determine the efficacy of PDT in palliative advanced rectal cancer. Six patients with advanced rectal cancer were treated with PDT after Photofrin II photosensitization (32). In 2014, Huggett et al. used verteporfin in the PDT treatment of pancreatic cancer in fifteen inoperable patients. The study results showed that the use of PDT was possible and safe (257). In 2019, DeWitt et al. used a combination of endoscopic ultrasound and photodynamic therapy EUS-PDT to treat pancreatic cancer. He used this innovative method to treat a group of 12 patient (77). Berr et al. used PDT for inoperable bile duct cancer in 23 patients and obtained improved palliation and extended survival (258).

##### 3.3.2.5 PDT in bladder cancer

Clinical PDT was firstly conducted by Jocham et al. in Germany to study bladder carcinoma in a group of fifteen patients in 1989. The PDT study resulted in the effective treatment of nine patients with no tumor recurrence (18). Kriegmair et al., who was a German researcher, also conducted studies used PDT for patients with bladder cancer with high efficiency of treatment (259). PDT using Photofrin^®^ provides good results in the treatment of papillary bladder cancer. Photodynamic diagnostics using 5-ALA also plays a significant role in urology for treatment of superficial bladder tumors. The photosensitizer 5-ALA is used in many different diseases. One type of disease is urinary tract anomalies. The results of the study by Waidelich et al. also showed significant effectiveness (260). In 2011, Hermann et al. used fluorescence diagnostics with HAL in patients diagnosed with a bladder tumor in 233 patients (261).

##### 3.3.2.6 PDT in gynecological cancer

PDT in gynecology is used to treat primary cancers of the female genital organs and metastatic tumors originating from other organs. The therapy covers pre-cancerous lesions and early invasive lesions of the vulva, cervix and ovary (262). Research shows that PDT gives very good results in the treatment of early cervical cancer and dysplastic changes in young women. *In vitro* studies conducted on ovarian cancer cell lines of various stages of advancement also indicate the effectiveness of the PDT method (263). In 2004, Löning et al. conducted clinical trials to assess the detection of fluorescence during metastasis of ovarian cancer in the second laparoscopic procedure following the intraperitoneal use of 5- ALA (264). Hillemanns et al. used 5-ALA in detecting metastases of ovarian cancer to the peritoneum in 26 patients (265).

##### 3.3.2.7 PDT in prostate cancer

PDT using Foscan^®^ and 5-ALA is used in the treatment of prostate cancer (266). In combination with bronchoscopy, PDT gives good results in the palliative unblocking of the respiratory tract in cases of advanced cancer. Photofrin^®^ or Telaporfin are used in the treatment of these tumors (267). Research in a group of 39 patients with prostate cancer have shown that PDD using protoporphyrin IX induced with 5-aminolevulinic acid (P-PIX) is effective (268). Trachtenberg et al. conducted studies on the vascular photosensitizer padoporfin and the results of the study confirmed the clinical potential of PDT in the treatment of prostate cancer recurrence (269). In 2013, Azzouzi et al. used PDT in prostate cancer clinical research for Eighty five patients. All patients were treated with the PDT method showed the effectiveness of the method and low side effects (270).

##### 3.3.2.8 PDT in esophageal cancer

Another example of clinical research was reported by McCaughan. The author enrolled 40 patients with esophageal cancer undergoing PDT into the study. The therapeutic goal was to enable patients to swallow more efficiently. The results of therapy were assessed based on the size of the tumor before and after therapy and the size of the minimum diameter of the opening in the esophagus. According to study reports, after therapy, the tumor shrank by an average of 1 cm and the diameter of the esophagus increased by an average of 3 mm, which was a significant therapeutic achievement (271). McCaughan et al. used PDT to treat esophageal cancer. The study was a prospective study and lasted 12 years. After the therapy, the authors noticed minor complications that did not pose a threat to the patient’s health or life. Based on the results, it can be concluded that PDT is a substitute method for the treatment of esophageal cancer (272). In 1999, Overholt et al. performed PDT treatment in patients with esophagus and Barrettís dysplasia and obtained the results of 80% of the treated cases (189). In 1997, Scheider et al. used PDT for the treatment of esophageal tumor in 4 patients with obstructive adenocarcinomas of the distal esophagus (273). Ophthalmology is another field of medicine in which one of the therapeutic methods used is PDT. Subfoveal choroidal neovascularization is one of the examples of ophthalmological diseases. Schmidt-Erfurth et al. are the authors of a study in which patients with this type of disease were treated with PDT. The applied photosensitizer was a benzoporphyrin derivative. The effectiveness and changes induced after PDT were assessed using fluorescein angiography. After the study, no fluorescein leakage was observed in most patients (274).

#### 3.3.3 PDT in dentistry

**Table 5** shows photosensitizers used in dentistry.

**Table 5:** PS application in dentistry.

PS application in dentistry	
**Photosensitizer**	**Current clinical state**
**5-ALA**	**clinical**
**ester methyl aminolevulinate (MAL)**	**clinical**

The concept of using PDT in the treatment of microbial infections in endodontics seems interesting. It is based on the exposure of microorganisms to exogenous or endogenous photosensitizer molecules and then the use of light with wavelengths in the red/near infrared region, which causes the formation of reactive oxygen species and, consequently, the death of microorganisms. This method may be particularly important in the case of drug-resistant microorganisms. After completing the canal preparation, the clinical procedure involves inoculating the canal with PS solution. This solution is left in the canal for a specified period of time, which is 60 seconds. It is particularly important to ensure adequate moistening, which allows direct contact of bacteria with the photosensitizer. The purpose of this stage is to enable contact of the solution with bacteria and diffusion through the biofilm structures. Then, a light emitter is inserted into the root canal and each canal is illuminated for 30 seconds. It has been proven that non-invasive PDT is able to disrupt the biofilm matrix, which facilitates comprehensive disinfection. Similar antibacterial properties of PDT are used in caries, as well as oral surgery and periodontology, where this method is an alternative in the treatment of alveolar bone inflammation, post-extraction pain, inflammation of tissues around the implant, local periodontal infections, and oral lichen planus (11, 91, 93). Photodynamic antimicrobial chemotherapy (PACT) has demonstrated its effectiveness in combating bacterial, fungal, parasitic and viral infections. It is worth noting that the lack of any genotoxic or mutagenic activity in the case of PDT is a key factor that ensures long-term safety during treatment. According to available research, in patients with mild to moderate periodontitis, the combination of PDT with scaling and root planning (SRP) may result in significantly greater clinical improvement than SRP alone. In patients with periodontitis with rapid progression and severe severity, photodynamic therapy improves the clinical condition, but should not be used as a substitute for amoxicillin and metronidazole (275). PDT causes a significant reduction of bacteria, but their complete elimination is usually not achieved (276–287). It is also important to use the most selective photosensitizers possible, because killing the entire physiological flora of the oral cavity exposes patients to infections with opportunistic pathogens (276).

#### 3.3.4 Combination of PDT with other types of anticancer therapy

**Figure 4:**
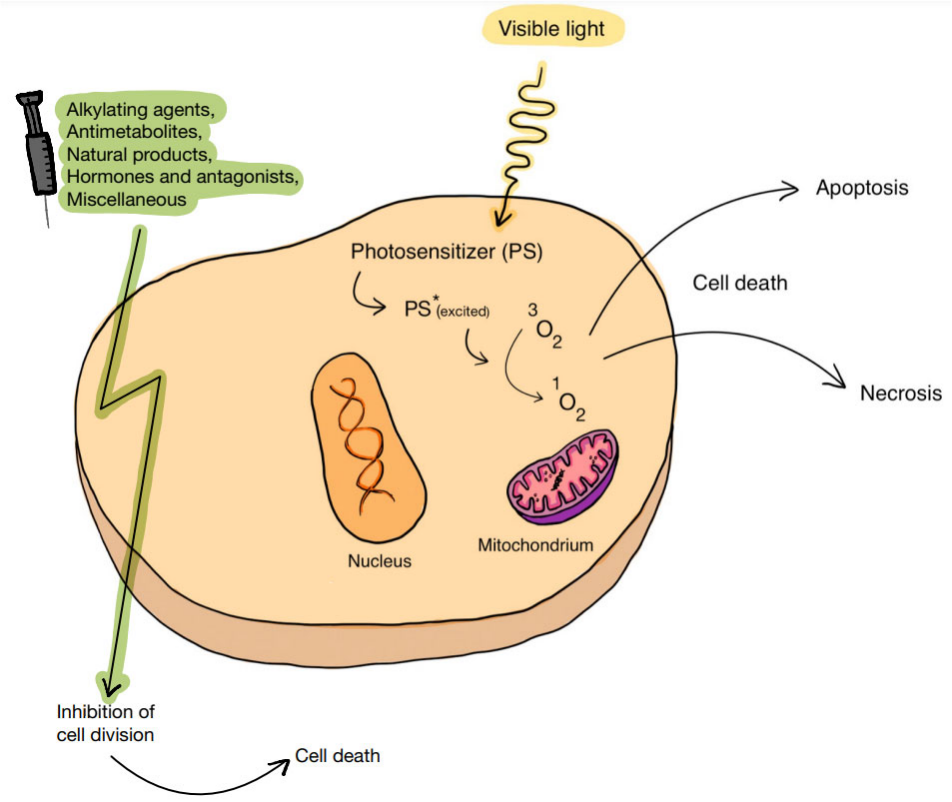
The cellular effect of PDT combined with chemotherapy. By generating ROS, an introduced PS can lead to cell death. The association of PDT with chemotherapy, which includes the use of various substances, including alkylating drugs, antimetabolites, natural products, and hormones and antagonists, can act favorably on the effect of anticancer therapy.

PDT, apart from its independent role in fighting cancer, can be combined with other therapeutic methods. Due to frequent drug resistance, numerous side effects (including serious systemic side effects), low bioavailability, lack of effectiveness in the treatment of heterogeneous tumors and the potential to stimulate tumor progression, stand-alone chemotherapy often does not produce satisfactory results. Therefore, it is important to combine PDT and chemotherapy in order to increase therapeutic effectiveness and at the same time reduce toxicity. An additional element of therapy may be the use of a drug delivery system (DDS), which may increase the bioavailability and safety of the drug. Currently, research is underway on various nanocarriers that jointly deliver chemotherapeutics and photosensitizers. **Figure 4** shows schematic summary of PDT combined with chemotherapy.

**Figure 5:**
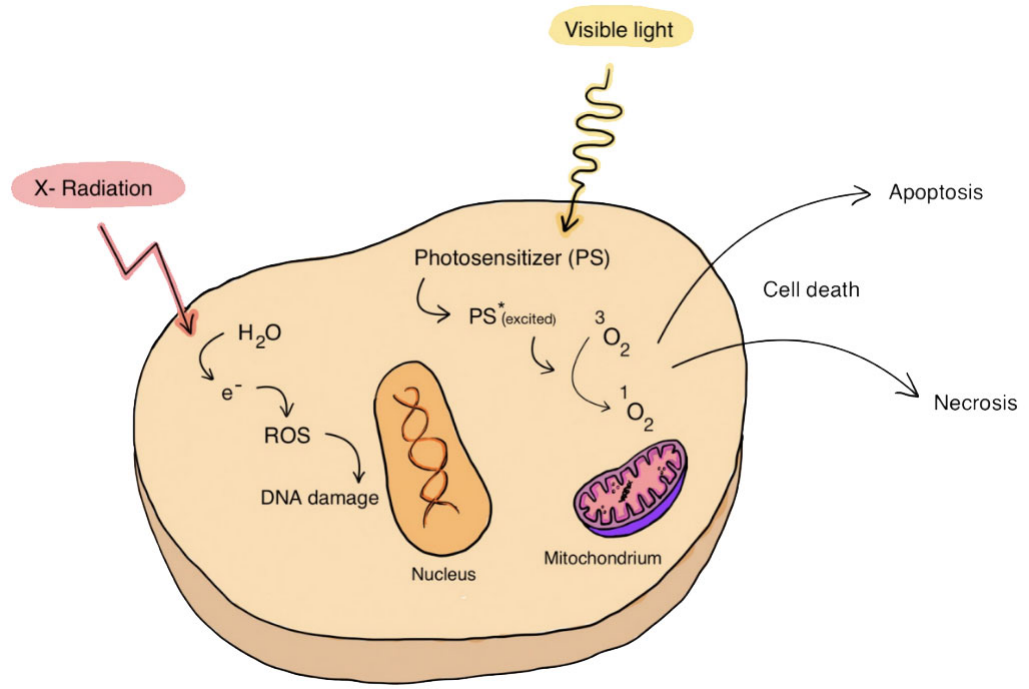
PDT combined with radiation therapy. The association of PDT with radiation therapy, includes the use of X-rays that can lead to the generation of free radicals, can act favorably on the effect of anticancer therapy.

Another local cancer treatment method is radiotherapy, which usually produces positive therapeutic effects. By combining PDT with radiotherapy, the effectiveness of tumor ablation can be dramatically increased. **Figure 5** shows schematic summary of PDT combined with radiation therapy.

A method of anticancer treatment that has been gaining popularity in recent years is gene therapy, which is safer and has fewer side effects than regular chemotherapy and radiotherapy. By using near-infrared (NIR) light, it is possible to deliver siRNA and antisense oligonucleotides (ASO) in the form of spherical nucleic acid (PSNA), which will be cleaved at the target site (cancer cells). By using PDT, locally released siRNA and ASO can inhibit the expression of hypoxia-inducible factor-1 (HIF1) and Bcl-2 protein, which ultimately inhibits tumor growth. The combination of surgery with PDT is based primarily on the use of fluorescent properties of some photosensitizers, which provides a visual aid for the surgeon (97). Standard fluorescence-guided surgery (FGS) uses fluorescent contrast agents such as indocyanine green (ICG), 5-aminolevulinic acid (5-ALA), and methylene blue (MB), which can be successfully used as photosensitizers in photodynamic therapy. Photodynamic diagnostics (PDD) is safer compared to conventional imaging methods, and thanks to the use of near infrared (NIR) light, it does not change the appearance of the surgical field. By combining FGS with PDT, the dual role of contrast agents allows easy and relatively accurate removal of the tumor by the surgeon, and at the same time allows for the removal of residual tumor fragments (277). Moreover, photodynamic therapy is significantly related to immunotherapy. PDT, by releasing tumor-associated antigens (TAAs) and danger-associated molecular patterns (DAMPs) from tumor cells, results in the body’s immune response (93). Another example of pilot studies related to PDT is DNA flow cytometry analysis. In a study by Foultier et al., cellular DNA content was analyzed during the cell cycle using flow cytometry. The analysis showed that tumor destruction occurred in 52% of the examined patients. According to the authors, the applied method has a destructive effect on tumor cells and blood vessels (278). In 2015, Kanick et al., demonstrated the effect of light on optical measurements of PpIX concentration and concluded that PpIX can be used as an objective predictor of response to ALA-PpIX PDT (279).

In Poland in 2013, Maździarz et al. compared vulvar lesions using two 5-ALA concentrations to assess results of histological examinations. All results showed a high correlation between the PDD method and vulvar lesions with the accuracy of histological examination (280).

### 3.4 Immunological aspects of PDT

**Figure 6:**
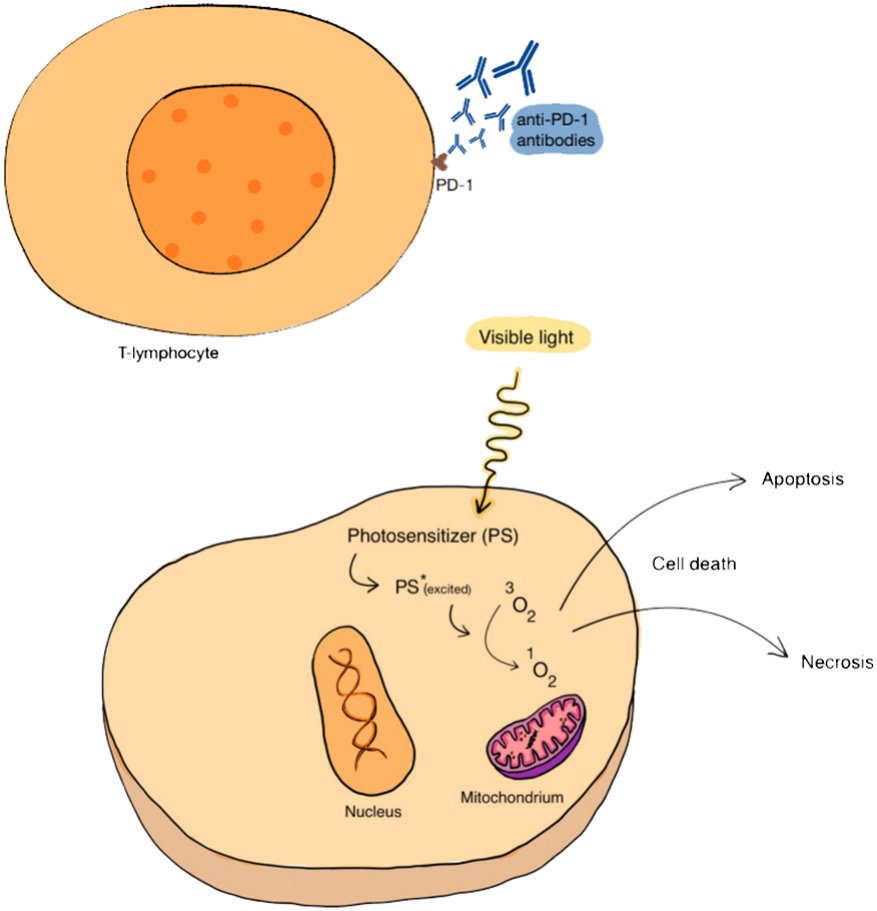
PDT combined with immunotherapy. The association of PDT with immunotherapy, which may include, among other things, the use of an anti-PD-1 antibody directed against receptors on T lymphocytes, has a beneficial effect on the effect of anticancer therapy.

Recent studies have shown that the combination of photodynamic therapy with immune checkpoint inhibitors, e.g. antibodies against programmed death 1 (anti-PD-1) or antibodies against programmed death 1 ligand (anti-PD-L1), may be used in anticancer treatment (281, 282). Under the influence of hypoxia-inducible factor 1α (HIF-1α), there is an increase in the expression of PD-L1, which is captured by PD-1 receptors located on activated cytotoxic T lymphocytes (CTL). Ligand binding inhibits the immune response (283). The synergistic mechanism of action of PDT with talaporfin sodium (TS-PDT) and blocking the anti-PD-1/PD-L1 pathway has been shown to influence cell death *in vitro* and tumor suppression *in vivo* in a mouse model. Sasaki et al. noted that tumor suppression also occurs on the non-irradiated side. Neoantigens released from damaged cells are captured by dendritic cells (DCs), presented to T lymphocytes, which become activated, move, infiltrate the tumor, recognize specific and tumor cells and kill them. The release of antigens is influenced by TS-PDT, while the use of anti-PD-1 antibodies induces the killing of tumor cells by T lymphocytes (282). **Figure 6** shows schematic summary of PDT combined with immunotherapy.

Recent research has revealed several mechanisms responsible for the body’s immune response stimulated by photodynamic therapy. It turns out that calreticulin (CRT), located in the endoplasmic reticulum (ER), moves to the surface of the cell membrane after PDT, giving a signal to the cells of the immune system to trigger an immune response. In addition, the expression of the NF-κB transcription factor, heat shock protein-70 and many cytokines (including IFN-γ, IFN-α) is increased and the mutation of antigen-presenting cells (APC) and Tc lymphocytes is promoted to colonize the tumor areas. Additionally, neutrophils are recruited and then macrophages infiltrate, while simultaneously stimulating the production of a specific tumor cell antigen (homogenate) (1, 95). As immunogenic cell death (ICD) occurs, further consequences occur. In addition to the exposure of CRT and heat shock protein (HSP) on the cell surface, adenosine triphosphate (ATP) and high mobility group 1 protein (HMGB1) are released from the cell (95). These substances act as molecular structures associated with damage (DAMPs/alarmins) (281). ATP initiates the recruitment of APCs in the tumor bed, CRT facilitates the phagocytosis of dead cells, HMGB1 promotes the presentation of numerous tumor-associated antigens (TAAs) to APCs, and HSPs mediate the maturation and migration of dendritic cells (DCs). Secreted pro-inflammatory cytokines accelerate the maturation of APCs. Therefore, ICD serves as a bridge between cancer immunotherapy and PDT (95). Another drug, ripasudil, an inhibitor of Rho kinase (ROCK), a GTPase that regulates the organization of the actin cytoskeleton, enhances phagocytic activity and the ability to process antigens by antigen-presenting cells (APCs), and in additional combination with an anti-PD-1 drug and photodynamic therapy with a nanophotosensitizer embedded in Ce6 (FIC-PDT) causes a systemic, long-lasting anti-tumor response of melanoma cells (284). The combination of PDT with the COX-2 inhibitor (isoenzyme of normal cyclooxygenase, found, among others, in cervical cancer cells) - celocoxib, significantly improves treatment results (285).

The undoubted advantage of PDT is its high selectivity and specificity, which reduces the cytotoxic effect and damage to normal cells, as is the case, among others, in radiotherapy or chemotherapy (131). PDT is a highly accurate method that prevents disease recurrence (286). Moreover, this method is comfortable and leaves virtually no scars, and apart from protection against light, it does not require any special precautions (287). In addition to the obvious advantages of PDT in cancer, some disadvantages hinder the clinical dissemination of this treatment method. The skin may have different physical properties (such as absorption, transmission, scattering, reflection of light), which affect the different generation of PpIX and individual differences, and even within different locations in the same patient (96). PDT may also be ineffective in hypoxic areas (due to the use of ROS in the PDT mechanism). Tumor hypoxia leads to attenuation of PDT, the effect of which is oxygen-dependent, and oxygen consumption in PDT aggravates tumor hypoxia, leading to a vicious circle (288). Tumor hypoxia promotes the proliferation of immunosuppressive cells (including M2-type macrophages), which further promotes tumor development and relapses (95). Moreover, the effectiveness of PDT is limited in deep-seated or deeply infiltrating tumors. This is due to the short wavelength of light (400-700nm), which limits penetration into tissues (289). Additionally, too high PS concentration causes aggregation-induced quenching (ACQ), which weakens the optical properties of PS, and its systemic administration may result in abnormal distribution and accumulation in normal tissues, causing a phototoxic effect (290–292). Thanks to the advancement of nanomedicine, the possibilities of improving the effectiveness of PDT seem promising. The problem of tumor hypoxia may be solved by self-oxygen strategies. Emerging biomaterials and therapeutics to alleviate hypoxia include:

-nanoparticles that produce oxygen by activating H_2_O_2_ (HAOP NP), e.g. manganese dioxide (MnO_2_), which decomposes and catalyzes the reaction of intracellular H_2_O_2_, gold nanocates (AuNC), which are, however, potentially toxic, catalase (CAT), which is an endogenous antioxidant enzyme.

-oxygen-carrying nanoparticles are particularly important in the case of hypoxic tumors in which the O_2_ level is too low, these include, among others: hemoglobin (Hb), combined with human serum albumin (HSA), which provides the complex with *in vivo* stability and biocompatibility, as well as an oxygen-carrying nanoperfluorocarbon (nanoPFC),

-nanoparticles that eliminate the physical barrier that hinders the diffusion of oxygen

-immunosuppressive tumor microenvironment (TME) contains an abnormal, dense extracellular matrix (ECM), which impedes the diffusion of oxygen and also reduces its supply by pressing on blood vessels. The use of hyaluronidase (HAase), which hydrolyzes cross-linked hyaluronic acid (HA), can facilitate oxygen access to the tumor.

-nanoparticles that reduce oxygen consumption by cancer cells, e.g. metformin (Met), which, by inhibiting the mitochondrial respiratory chain, reduces oxygen consumption and indirectly reduces tumor hypoxia.

The limited depth of light penetration is attempted to be overcome by using UCN - nanometer-sized materials that transform low-energy light into high-energy light (95). Moreover, in 2016, G. Tortora et al. developed a capsule with a length of 27 mm and a diameter of 14 mm, equipped with 8 LED diodes, which, by emitting appropriate blue (405 nm) and red (625 nm) light, excited naturally occurring photosensitizers in the Helicobacter pylori bacterium, which led to consequently, to kill this Gram-negative bacterium (93, 95). Recent research includes the use of similar medical microdevices in the treatment of several types of gastrointestinal cancers (293). Moreover, the use of ultrasound activating PS instead of a standard light source seems interesting (97). Sonodynamic therapy (SDT) is an interesting alternative to PDT, which, with the appropriate combination of frequency, intensity, duty cycle and duration of use of ultrasound and drugs that increase light sensitivity, can lead to cell death. However, further research is necessary to help translate *in vitro* studies into living organisms (294).

Modern nanocarriers can precisely distribute PS, leading to their accumulation only within cancer cells. Co-delivery of PS and immunomodulators may result in optimal cancer immunotherapy. Intelligent tumor microenvironment (TME)-responsive nanoplatforms reduce phototoxicity by controlling premature leakage of PS into the circulation and at the same time accelerate its release to overcome the ACQ effect. Nanoorganic metal particles (nMOF) have porous structures, which allows them to provide high PS charges, easy oxygen diffusion, and overcome quenching caused by aggregation. The latest research includes complex, intelligent nanoparticles that respond to TME conditions and, combining the above features, provide optimal conditions for effective photodynamic therapy (95).

An acute side effect of local PDT may be pain, most likely of neurogenic and inflammatory origin, which is difficult to control pharmacologically. Currently, due to the optimization of treatment regimens, it is not a common limiting factor (94). Some patients may complain of tingling, burning or itching during PDT, as well as local erythema or swelling, or even visible hives. However, this is an effect expected in photodynamic therapy (94, 295, 296). Rarely, undesirable effects of PDT include hypo- or hyperpigmentation changes, scars or allergic reactions (94).

#### 3.4.1 Antibacterial and antifungal PDT

A new direction in the development of PDT is photodynamic inactivation of microorganisms (PDI). Bacteria, viruses and fungi can be inactivated by combining non-toxic dyes with harmless visible light. It is an interesting alternative to antibiotics and antiviral drugs, which can be used in medicine, veterinary medicine, agri-food areas, and sewage treatment plants. In the context of the constantly growing resistance of bacteria to bactericidal and bacteriostatic drugs, it seems particularly important to find new methods of combating pathogenic microorganisms. The limitation is the shallow depth of light penetration, which makes the treatment of infection difficult (11). Promising results of the antibacterial and antifungal activity of PDT were obtained using: rose bengal, methylene blue, cationic porphyrins and fullerenes. Rose Bengal turned out to be bactericidal against the Streptococcus genus, and methylene blue was toxic to the Candida strain fungi (297, 298).

#### 3.4.2 Vascular PDT

In medical practice, PDT has been used also to target the vascular endothelium (“vascular target”) (299).

PDT with the use of Verteporfin allows stopping the degenerative process of wet degeneration. It causes obliteration (closure) of pathological vessels (300, 301). In 2003, Fossarello et al. to treat corneal neovascularization (CNV) used verteporfin. They obtain positive effects however, multiple sessions are required (302). In the study by Rockson et al. used motexafine lutetium as a photosensitizer in patients with atherosclerotic peripheral arterial insufficiency. Therapy was well tolerated over the entire dose range of motexafine lutetium and light tested. There were no procedural complications directly attributable to experimental photoangioplasty. Rare side effects were limited to transient paresthesia and minor, transient, self-limited skin eruptions, and no phototoxic effects were observed. Researchers suggest that PDT with motexafine lutetium is a promising alternative intervention for the treatment of flow-limiting atherosclerosis (303). Kereiakes et al. performed phototherapy with Motexafin lutetium (MLu) in patients previously undergoing percutaneous coronary intervention with stent placement. Motexafin lutetium was administered intravenously to 79 patients 18-24 hours before the procedure, and photoactivation was performed after initial balloon dilation and before stent placement. MLu was well tolerated without serious dose-limiting toxicities, and the most frequently observed adverse events were peripheral paresthesia and rash, but these were generally mild and self-limiting. No unfavorable angiographic results were attributed to phototherapy. A range of optimal dosing regimens was assessed, where an MLu dose of 2–3 mg/kg and a light fluence of 100–400 J/cm-fiber were safe and well tolerated (304). Further clinical trials are necessary to evaluate the therapeutic potential and exclude any significant toxicity of PDT with Motexafin (40).

### 3.5 Methods of enhancing the effects of PDT

#### 3.5.1 PDT combined with chemotherapy increasing effect and decreasing toxicity

Dual pH response characteristic of mesoporous silica nanoparticles were achieved by Xuemei Yao et al. (305) by acid-sensitive polyethylene glycol tetraphenyl porphyrin zinc (Zn-PO-CA-PEG) conjugates. They are acid-sensitive by cis-anhydride (CA) part and at extracellular pH (~6.8), leaving the Zn-PO with a positive amino charge on its surface, remarkably promoting cellular internalization (306). Zhou et al. prepared a photoactivated Pt (IV) prodrug polymeric PtAIECP. The chemotherapy drug doxorubicin (DOX) is wrapped in the PtAIECP to form the composited nanomaterial (PtAIECP@DOX), which have self-detection drug release performance and PDT-based synergistic therapy system. The effects of PtAIECP@DOX on prodrug activation, drug release, and synergistic therapy are verified *in vitro* and *in vivo* experiments (97, 307).

#### 3.5.2 PDT combined with radiation therapy

Liu Zhiyang et al. (308) synthesized four polymers based on a triphenylamine-azafluorenone core that exhibit different photophysical properties and excellent biological applications. Cationization is an effective strategy to improve ROS generation and PDT efficiency of PSs. Cationized mitochondria-targeted PS shows a higher PDT efficiency than the non-ionized alternatives. Due to the AIE and ISC effects, the fluorescence intensity and ROS generation of AIE PSs increase simultaneously from the molecularly dispersed state to the aggregated state, which makes image-guided PDT possible without a complicated chemical process. In addition, several AIE PSs can also be employed as radiotherapy sensitizers. Compared to monotherapy, the combination of PDT plus radiation therapy dramatically boosts the efficacy of tumor ablation. This work provides a valuable exploration for improving PDT efficiency through the molecular design of PSs.

#### 3.5.3 PDT combined with immunotherapy

PDT can effectively trigger the release of tumor-associated antigens (TAAs) and danger-associated molecular patterns (DAMPs) from tumor cells, which would contribute to the induction of strong immune responses (309). Currently, several nanoplatforms are known that increase blood flow and reduce the degree of hypoxia in the tumor tissue environment, thus increasing the therapeutic effects of PDT (310). Normalizing blood vessels also significantly facilitates the infiltration of immune cells. Another example are nanoplatforms simulating NK cells that can penetrate the blood-brain barrier (311). The authors repeatedly use composites such as polymers and nanoparticles. Overall, PDT combined with immunotherapy can not only improve immune cell activity but also reduce immune escape of cancer cells (3, 86, 312, 313). The use of PDT in the clinic still requires significant effort and overcoming technological difficulties. Therefore, work on the development of the PS-based PDT method in cancer treatment continues. The conducted research focuses mainly on the following three improvement strategies, i.e. (1) optimization of a PS that would be resistant to hypoxic conditions and produce ROS efficiently even in the anaerobic tumor environment; (2) design of a tumor-targeting nanocarrier and activation of PS by the tumor microenvironment; and (3) increasing the penetration depth of the excitation light for PS, for example through chemiluminescence and bioluminescence strategies.

## 4 Conclusions

PDT is currently a rapidly developing treatment method which, when using an appropriate PS and a light beam, can lead to the formation of reactive oxygen species and thus to the death of the marked cell. Currently, it is used not only in anticancer therapy, but also in the treatment of non-cancer skin diseases, inflammations and infections caused by various microorganisms. Some diseases have developed treatment regimens, others require further research to consider whether the benefits of the therapy exist and whether they outweigh the possible side effects. Nowadays, PDT on skin cancer has been widely promoted in the department of Dermatology in China. The large population and relatively weaker awareness of UV protection have resulted in a correspondingly large number of skin cancer patients. This has given PDT great prospects for application, but also poses many challenges. Combining PDT with other anticancer therapy methods, such as chemotherapy, radiotherapy, gene therapy, surgery and immunotherapy, seems to be promising, and the latest studies confirm their synergistic effect, while reducing the risk of side effects, including severe pain. Despite the many advantages of PDT, there are still problems that may impede the proper course of therapy. These include, among others: various physical properties of the skin, oxygen availability for the tumor, limited light penetration into tissues, ACQ effect associated with too high PS concentration in the cell and others. Thanks to the advancement of nanomedicine, some of them have already been overcome, others require further research. It is justified to develop modern strategies in photodynamic therapy that will allow the development of this field and, consequently, lead to a decrease in mortality due to many diseases affecting modern people, as well as improve the quality of their life. Relatively simple in its mechanism, photodynamic therapy may prove to be a breakthrough method of treating cancer and more. PDT in oncology is a very promising technique, however, still a small number of hospitals and cancer centers throughout the country have skilled doctors and the machines needed to perform PDT. The method has selectivity of action as PDT in tissue is limited to the irradiated area. Treatment protocols are simple to follow and there are less complications when using PDT. Tissues regenerate faster, which ensures a very good cosmetic effect. The method is minimally invasive towards deeper structures and allows the function of the treated organ to be preserved. It gives good results in the treatment of primary lesions, as well as in palliative treatment or as a complement to surgery, radio- and chemotherapy. Further development of this field may be of key importance in the medicine of the future. PDT is a relatively new treatment option and usually performed as an outpatient procedure with high effective rate and minimal side effects. PDT is most commonly used to treat precancerous skin conditions such as actinic keratosis, as well as basal cell and squamous cell carcinomas. Recently, studies have focused on the efficacy comparison between PDT and different treatment methods or the improvement of efficacy brought by the combined application. Many studies have also focused on the continuous upgrading of photosensitizers. More research is urgently needed to determine PDT’s effectiveness in treating various types of skin cancer and its long-term outcomes.

## Author contributions

DA: Conceptualization, Data curation, Formal analysis, Investigation, Methodology, Resources, Software, Supervision, Validation, Visualization, Writing – original draft, Writing – review & editing. KR: Conceptualization, Data curation, Formal analysis, Investigation, Methodology, Resources, Software, Validation, Visualization, Writing – original draft, Writing – review & editing. AM: Conceptualization, Data curation, Formal analysis, Investigation, Methodology, Resources, Software, Validation, Visualization, Writing – original draft, Writing – review & editing. KD: Conceptualization, Data curation, Formal analysis, Investigation, Methodology, Resources, Software, Validation, Visualization, Writing – original draft, Writing – review & editing. RW: Conceptualization, Data curation, Formal analysis, Methodology, Resources, Validation, Funding acquisition, Writing – original draft, Writing – review & editing. GC: Conceptualization, Data curation, Formal analysis, Methodology, Resources, Validation, Funding acquisition, Investigation, Writing – original draft, Writing – review & editing. AK-K: Conceptualization, Data curation, Formal analysis, Methodology, Resources, Validation, Funding acquisition, Investigation, Writing – original draft, Writing – review & editing. DB-A: Conceptualization, Data curation, Formal analysis, Investigation, Methodology, Resources, Software, Validation, Visualization, Writing – original draft, Writing – review & editing.
